# HDAC6 Signaling at Primary Cilia Promotes Proliferation and Restricts Differentiation of Glioma Cells

**DOI:** 10.3390/cancers13071644

**Published:** 2021-04-01

**Authors:** Ping Shi, Lan B. Hoang-Minh, Jia Tian, Alice Cheng, Reemsha Basrai, Neil Kalaria, Joseph J. Lebowitz, Habibeh Khoshbouei, Loic P. Deleyrolle, Matthew R. Sarkisian

**Affiliations:** 1 Department of Neuroscience, University of Florida College of Medicine, Gainesville, FL 32610, USA; shiping@ufl.edu (P.S.); tian.jia@ufl.edu (J.T.); acheng1@ufl.edu (A.C.); reemshab@usf.edu (R.B.); nk2850@ufl.edu (N.K.); lebowitz@ohsu.edu (J.J.L.); habibeh@ufl.edu (H.K.); 2 Preston Wells Center for Brain Tumor Research, University of Florida College of Medicine, Gainesville, FL 32610, USA; Lan.Hoang-Minh@neurosurgery.ufl.edu (L.B.H.-M.); Loic.Deleyrolle@neurosurgery.ufl.edu (L.P.D.); 3 Department of Neurosurgery, University of Florida College of Medicine, Gainesville, FL 32610, USA

**Keywords:** histone deacetylase 6, primary cilium, glioblastoma, ARL13B, alpha-tubulin

## Abstract

**Simple Summary:**

Glioblastoma is the most common and lethal brain tumor in adults because it becomes resistant to virtually every treatment. Histone deacetylase 6 (HDAC6), which is located primarily in the cytoplasm, has a unique role in promoting the disassembly of cells’ primary cilium, a non-motile “antenna” that must be broken down before cells can progress through the cell cycle. The role of HDAC6 and its function in gliomas have not been investigated with respect to tumor cell cilia. We have found that inhibitors of HDAC6 cause rapid and specific changes inside glioma cilia, reducing tumor cell proliferative capacity and promoting cell differentiation. Importantly, the HDAC6 inhibitors did not affect the proliferation or differentiation of glioma cells that we genetically modified unable to grow cilia. Our findings reveal a conserved and critical role for HDAC6 in glioma growth that is dependent on cilia.

**Abstract:**

Histone deacetylase 6 (HDAC6) is an emerging therapeutic target that is overexpressed in glioblastoma when compared to other HDACs. HDAC6 catalyzes the deacetylation of alpha-tubulin and mediates the disassembly of primary cilia, a process required for cell cycle progression. HDAC6 inhibition disrupts glioma proliferation, but whether this effect is dependent on tumor cell primary cilia is unknown. We found that HDAC6 inhibitors ACY-1215 (1215) and ACY-738 (738) inhibited the proliferation of multiple patient-derived and mouse glioma cells. While both inhibitors triggered rapid increases in acetylated alpha-tubulin (aaTub) in the cytosol and led to increased frequencies of primary cilia, they unexpectedly reduced the levels of aaTub in the cilia. To test whether the antiproliferative effects of HDAC6 inhibitors are dependent on tumor cell cilia, we generated patient-derived glioma lines devoid of cilia through depletion of ciliogenesis genes ARL13B or KIF3A. At low concentrations, 1215 or 738 did not decrease the proliferation of cilia-depleted cells. Moreover, the differentiation of glioma cells that was induced by HDAC6 inhibition did not occur after the inhibition of cilia formation. These data suggest HDAC6 signaling at primary cilia promotes the proliferation of glioma cells by restricting their ability to differentiate. Surprisingly, overexpressing HDAC6 did not reduce cilia length or the frequency of ciliated glioma cells, suggesting other factors are required to control HDAC6-mediated cilia disassembly in glioma cells. Collectively, our findings suggest that HDAC6 promotes the proliferation of glioma cells through primary cilia.

## 1. Introduction

Glioblastoma (GBM), the most common and lethal aggressive brain tumor in adults, usually becomes refractory to standard-of-care treatments that include surgery, irradiation, and temozolomide (TMZ) chemotherapy. Histone deacetylase 6 (HDAC6) is a therapeutic target in non-central nervous system (CNS) cancers that is receiving increasing scrutiny in GBM [1,2,3,4,5,6,7,8,9]. Compared to other HDACs, HDAC6 expression is disproportionately high in GBM [1,3,10]. Notably, HDAC6-specific inhibitors reduce the proliferation and viability of GBM cells in vitro [6,9,10,11] and in subcutaneous and orthotopic models of GBM [1,12]. Lentiviral-mediated knockdown of HDAC6 also slows tumor growth and promotes survival in an intracranial model of GBM [7]. There is an increasing number of reports of small molecule inhibitors of HDAC6 in development; some of these inhibitors (e.g., ACY-738, ACY-1083) have been shown to cross the blood–brain barrier [13,14], while others enhance the effects of TMZ and irradiation therapy in GBM cells in vitro [2,5,6,7,8,15,16]. Thus, there is a strong rationale for introducing HDAC6 inhibitors as adjunct therapies for malignant brain tumors, warranting in-depth analyses on the mechanisms by which HDAC6 regulates gliomagenesis.

Unlike the 17 other HDAC enzymes, HDAC6′s primary deacetylating targets are nonhistone substrates, particularly aaTub, which is a major component of the cell cytoskeleton [17,18]. HDAC6 also plays a key role in mediating the disassembly of primary cilia during the G0 or G1 cell cycle phases. Primary cilia are microtubule-based organelles typically containing an aaTub-positive axoneme that must be disassembled before cells can enter mitosis [19]. Aurora-A kinase, located around the ciliary basal body, phosphorylates HDAC6, stimulating its deacetylase activity on aaTub in the cilia, which in turn drives cilia disassembly and mitotic entry [20,21]. Thus, preventing cilia disassembly by blocking HDAC6 activity could arrest the division of cells within actively proliferating populations such as tumors. In cholangiocarcinoma cells, overexpression of HDAC6 reduces ciliogenesis, whereas knocking down or inhibiting HDAC6 prevents ciliary breakdown and increases the number of ciliated cancer cells [22]. Treatment with an HDAC6 inhibitor such as Tubastatin-A increases the frequency of ciliated cholangiocarcinoma cells and decreases their proliferation, and ablation of cilia by disruption of key ciliogenesis gene Intraflagellar transport protein 88 (*IFT88*) abolishes this antiproliferative effect [22]. Chondrosarcoma growth is similarly affected by HDAC6 inhibitors and their effects on cilia [23]. Together, these studies suggest that ciliated tumor cells may be more sensitive to HDAC6 inhibitors and raise an important and unanswered question as to whether HDAC6 signaling and promotion of tumor cell proliferation are dependent on cilia in glioma.

We previously reported that GBM biopsies and derived lines contain cells harboring aaTub+ primary cilia [24]. Over 90% of clones isolated from patient GBM cell lines produce ciliated cells [25], indicating that cilia may be more frequent in gliomas than has been previously described [26]. The goal of the present study was to examine whether the effects of HDAC6 inhibition on glioma cell proliferation are dependent on tumor cilia. In addition, because HDAC6 inhibitors disrupt gliomagenesis by converting the phenotype of actively dividing glioma cells to more differentiated states [5,7], we examined whether this differentiation is also dependent on the presence of cilia. Finally, we determined the effects of overexpressing HDAC6 on the frequency of ciliated glioma cells.

## 2. Results

### 2.1. HDAC6 Inhibitors Inhibit the Proliferation of Murine and Human Glioma Cells

HDAC6 inhibitors can significantly reduce glioma cell proliferation by disrupting the G2/M transition, increasing cell death, and promoting cell cycle exit and tumor cell differentiation [1,7,8]. We first confirmed that our patient-derived L0 (high grade), S3 (high grade), and S7 (low grade) glioma cell lines are sensitive to HDAC6 inhibitors by treating dissociated cells in serum-free media with different concentrations of 1215 and 738. After five days, 0.5 to 5 µM treatment with either drug significantly reduced the size of L0 and S7 (Figure S1A–G) and S3 gliomaspheres (Figure S1K), suggesting decreased tumor cell proliferation. After 24- or 48-h exposure of L0 cells to 1-µM 1215, flow cytometry analyses revealed significant increases in the number of tumor cells in the G2/M phase, a critical point in cell cycle control and differentiation, when compared to vehicle-treated cells (Figure S1H). The treatment also resulted in significantly enhanced tumor cell apoptosis, as indicated by the increased percentage of cells in the sub-G1 phase at both time points (Figure S1I). The proliferation of adherent murine KR158 glioma cells was also decreased following treatment with 5-µM 1215 or 738 but remained unchanged with a lower concentration (500 nM) of either drug (Figure S1J). Together, our results are consistent with recent findings and show that inhibitors of HDAC6 prevent the proliferation of glioma cells by promoting cell death and cell cycle arrest.

### 2.2. HDAC6 Inhibitors Rapidly and Differentially Alter Alpha-Tubulin Acetylation in Glioma Soma versus Primary Cilia

Next, we examined how 1215 and 738 affected aaTub expression in human and murine glioma cells. Consistent with other reports [20,21,27], we found that HDAC6 was expressed throughout cell somas (Figure S2). In S3 cells, we detected punctate clusters of HDAC6 around gamma-tubulin+ centrioles and ciliary basal bodies (Figure S2A–C)**,** and although we could find some rare HDAC6+ puncta colocalized with aaTub+ cilia axonemes (Figure S2D–I), HDAC6 was not readily detectable in cilia. A similar pattern was observed in S7 cells (Figure S2J–R). Nonetheless, we hypothesized that inhibiting HDAC6 activity might result in widespread increases in aaTub expression, including within the cilia.

**Figure 1:**
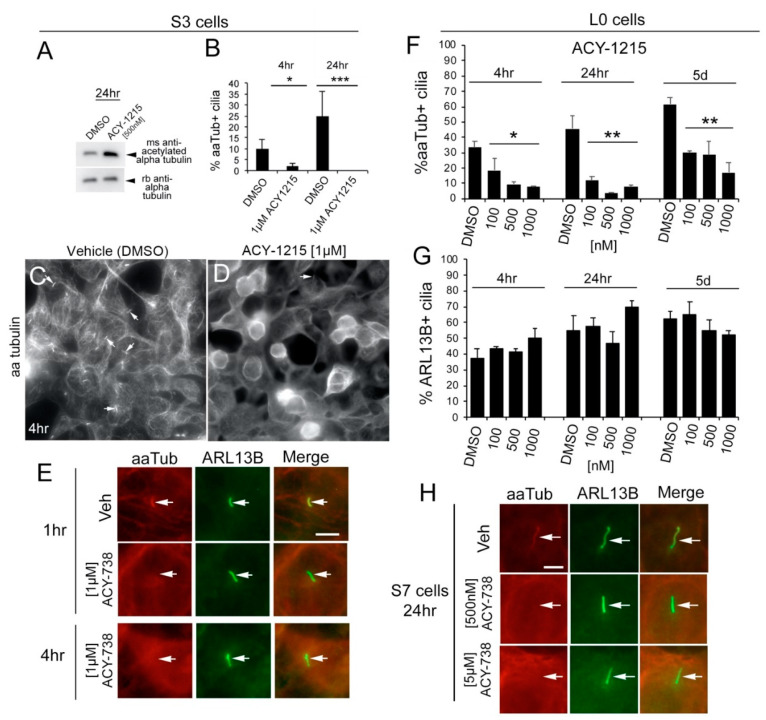
Treatment with histone deacetylase 6 (HDAC6) inhibitors triggers a rapid and sustained reduction of acetylated alpha-tubulin (aaTub) within ARL13B-positive glioma cilia. (**A**) S3 cell lysates 24 h after 1215 exposure. Western blot for aaTub and total alpha-tubulin. (**B**) Percentage of cells with aaTub+ cilia 4 or 24 h after 1-µM 1215 treatment compared to vehicle (equal volume of dimethyl sulfoxide (DMSO)). (**C**,**D**) S3 cells 4 h after 1-µM 1215 exposure immunostained for aaTub. Arrows point to aaTub+ cilia. (**E**) S3 cells 1 or 4 h after 1-µM 738, fixed and immunostained for aaTub (red) and ARL13B (green). aaTub readily colocalizes with ARL13B in vehicle-treated cells (top panels) but not in 738-treated cells (middle and bottom panels). (**F**,**G**) L0 cells treated with vehicle (DMSO) or indicated concentrations of 1215. Cells were fixed after 4 h, 24 h, or 5 days and immunostained for aaTub and ARL13B. Bar graphs show the % of aaTub+ cilia (**F**) and ARL13B+ cilia (**G**) for each drug/timepoint. (**H**) S7 cells treated with vehicle, 500-nM, or 5-µM 738, fixed 24 h later and immunostained for aaTub and ARL13B. Bars in E, H = 5 µm. * *p* < 0.05, ** *p* < 0.01, *** *p* < 0.001, ANOVA.

Indeed, lysates of S3 glioma cells treated with 1215 for 24 h showed robust increases in aaTub relative to total alpha-tubulin (Figure 1A). However, compared to vehicle-treated cells, it was unexpectedly difficult to find aaTub+ cilia across S3, L0, and S7 human glioma cells following treatment with either inhibitor. The presence of aaTub+ cilia on S3 cells was reduced after 4-h and 24-h exposure to 1215 (Figure 1B–D), and as early as 1 h after treatment with 738 (Figure 1E). Importantly, the decrease in aaTub+ in cilia was not indicative of cilia loss because cilia were present and identified by co-labeling with an antibody against ADP ribosylation factor 13B (ARL13B) (Figure 1E). We performed a dose and time-course experiment by treating L0 cells with vehicle or 100-, 500-, or 1000-nM 1215 and fixing cells after 4 h, 24 h, or 5 d for immunostaining for aaTub and ARL13B. We observed significant reductions in the percentage of aaTub+ cilia for each dose and time point (Figure 1F). However, 1215 did not significantly change the percentage of ARL13B+ cilia (Figure 1G). Similar decreases in aaTub+ cilia were observed after treatment of S7 cells with different concentrations of 738 (Figure 1H). The ARL13B remaining in the cilia following alpha-tubulin deacetylation could ensure ciliary membrane integrity and stability of the ciliary axoneme through binding of tubulin [28,29,30]. To assess whether HDAC6 inhibitors disrupted the microtubular structure of the ciliary axoneme, leading to the reduced aaTub observed by immunostaining, we examined the cilia of treated cells using TEM. Since 738 showed similar effects as 1215 (Figure 1E,H), we fixed and examined the ciliary structure of L0 cells 24 h after 5-µM 738 exposure; we found cilia with organized, longitudinally aligned arrays of microtubules along the axoneme (Figure S3A,B), suggesting that the ciliary microtubular structure was conserved following treatment with HDAC6 inhibitors.

**Figure 2:**
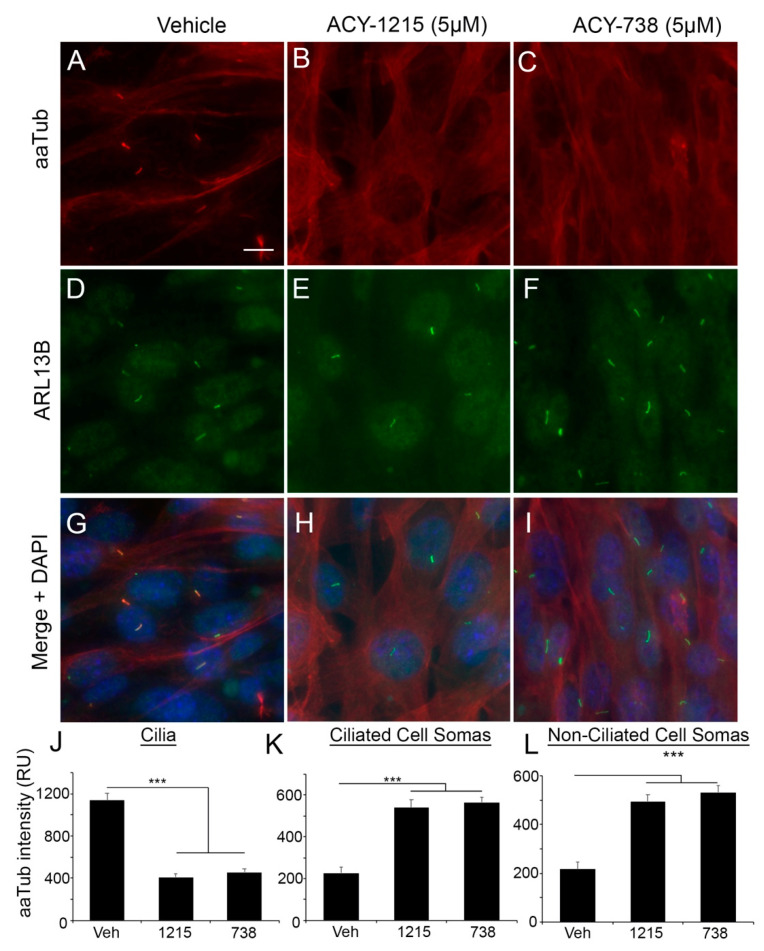
ACY1215 and ACY738 have differential effects on acetylated alpha-tubulin levels in murine glioma cell body and cilia. (**A**–**I**) Murine KR158 cells treated with vehicle, 5-µM 1215, or 5-µM 738 at 90% confluence for 24 h. Immunostaining for aaTub and ARL13B (arrow). (**J**) Quantification of background-corrected aaTub staining intensity of ARL13B+ cilia for each group. RU = relative units. (**K** and **L**) AaTub staining intensity of ciliated (**K**) and non-ciliated (**L**) somas. Bar in A = 10 µm. *** *p* < 0.001, ANOVA.

We also observed similar HDAC6 inhibitor-associated changes in mouse KR158 cells. Both 1215 and 738 treatment resulted in increased aaTub levels throughout the cell bodies when compared to vehicle-treated cells (Figure 2A–I). However, within cilia identified through ARL13B immunostaining, we observed weak expression of aaTub following treatment with either 1215 (Figure 2B,E,H) or 738 (Figure 2C,F,I). AaTub intensity was significantly lower in the cilia (Figure 2J) than in the cell bodies of ciliated or non-ciliated cells (Figure 2K,L). Altogether, these findings show that HDAC6 inhibitors induce a rapid but differential subcellular response in the degree of acetylation of alpha-tubulin in ciliated glioma cells.

### 2.3. Normal Murine Astrocytes Show Ciliary Responses to HDAC6 Inhibition Similar to Glioma Cells

We next examined whether the effects of HDAC6 inhibitors on glioma cilia extend to normal neural cell types that harbor aaTub+ and ARL13B+ cilia, such as radial glial cells and GFAP+ astrocytes [26,31,32,33]. We treated primary cultures of mixed cell lineages derived from fetal mouse midbrains and grown for 12–14 days in vitro with vehicle or 1-µM 1215 for 24 h and fixed and immunostained cells for GFAP, ARL13B, and aaTub. Compared to vehicle-treated astrocytes, 1215-treated astrocytes displayed significantly reduced levels of aaTub within their cilia (Figure S4A–F). These findings suggest that the effects of HDAC6 inhibition on ciliary aaTub are not glioma-cell specific.

### 2.4. HDAC6 Inhibition Stimulates an Increase in ARL13B+ Ciliated Glioma Cells

**Figure 3:**
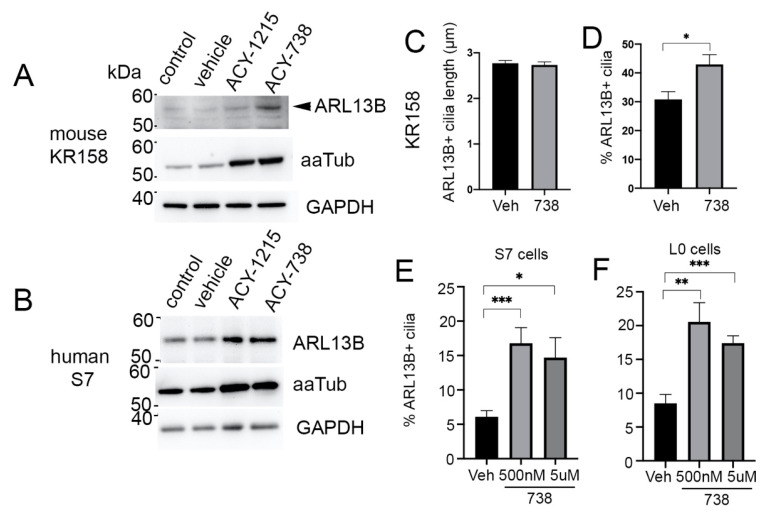
ACY738 treatment induces increased ARL13B protein levels and ARL13B-positive cilia frequency in glioma cells. (**A**,**B**) Murine KR158 (**A**) or patient-derived S7 (**B**) cells treated with vehicle or 5-µM 1215 or 738. Cells were harvested and lysed after 24 h, and protein lysates were Western blotted for ARL13B, aaTub, and GAPDH as a loading control. The 738 exposure induced a robust increase in ARL13B in KR158 cells, whereas both 1215 and 738 treatment increased ARL13B in S7 cells. (**C**,**D**) Length (**C**) and percentage (**D**) of ARL13B+ cilia in KR158 cells after vehicle (DMSO) or 5-µM 738 24-h exposure. * *p* < 0.05, Student’s *t*-test. (**E**,**F**) Percentage of ARL13B+ cilia in S7 (**E**) and L0 (**F**) cells after 24-h exposure to the vehicle (DMSO), 500-nM, or 5-µM 738. * *p* < 0.05, ** *p* < 0.01, *** *p* < 0.001, ANOVA.

Through Western blot analyses, we found that 5-µM 738 and 1215 promoted a similar increase in aaTub in murine KR158 cells, but 738 induced a larger increase in ARL13B levels than 1215 (Figure 3A). In human S7 cells, 5-µM 738 and 1215 promoted a similar increase in aaTub and ARL13B levels (Figure 3B). However, in normal mouse primary brain cultures, although aaTub levels were increased as expected, ARL13B levels remained unchanged after treatment with either inhibitor (Figure S4G). Therefore, although we found that HDAC6 inhibitors affected ciliary aaTub similarly in murine glia and human/murine glioma, Western blot analyses of ARL13B, a protein required for both ciliogenesis and cilia signaling, suggested there may be both inhibitor- and cell-type-specific differences in the ciliary response to HDAC6 inhibition. To investigate whether the 738-induced increase in ARL13B levels was due to an increase in cilia length, as ARL13B is involved in ciliary membrane extension [29], or an increase in the number of ARL13B+ ciliated cells, since 738 may prevent cilia disassembly, we examined both KR158 cell cilia length and percent of ARL13B+ ciliated cells 24 h after 5-µM 738 exposure. We found that the length of the cilia remained constant (Figure 3C), while the percentage of ARL13B+ ciliated cells significantly increased after the 738 treatment (Figure 3D). The 1215 exposure did not robustly increase ARL13B levels, cilia length, or percentage of cilia on KR158 (data not shown) or L0 cells (Figure 1G), suggesting that, while 1215- and 738-induced changes in ciliary aaTub are similar, there may be both HDAC6-inhibitor- and cell-line-specific effects on the presence of ARL13B+ cilia. Similar to KR158 cells, 738 induced a significant increase in the frequency of ARL13B+ cilia in S7 (Figure 3E) and L0 (Figure 3F) cells, but there did not appear to be a concentration-dependent increase in the number of cilia in either line. This finding suggests that there may be a limited number of ciliated glioma cells that can either form or arrest in the presence of inhibitors such as 738.

### 2.5. Preventing Cilia Formation Blocks the Antiproliferative Effect of HDAC6 Inhibitors on Glioma Cells at Low Drug Concentrations

**Figure 4:**
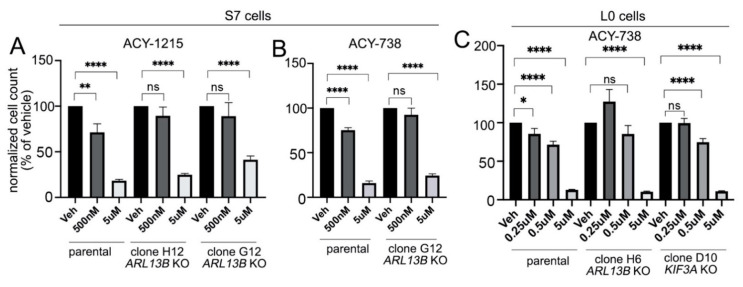
The ACY1215 and ACY738-induced decreases in glioma cell proliferation depend on inhibitor concentration and ARL13B and KIF3A/cilia. (**A**,**B**) Dissociated S7 parental cells or ARL13B KO clones were grown in 24-well plates for five days in vehicle or indicated concentrations of 1215 (**A**) or 738 (**B**). Bar graphs show the mean (±SEM) total cell counts (*n* = 8 wells/group) normalized as a percentage of vehicle. (**C**) Dissociated L0 control, *ARL13B*- or *KIF3A*-KO clones treated with vehicle or indicated concentrations of 738. Bar graph shows cell counts normalized to vehicle-treated controls. Data was statistically compared using ANOVA (* *p* < 0.05, ** *p* < 0.01, **** *p* < 0.0001).

We next examined if the antiproliferative effects of HDAC6 inhibitors are influenced by the presence of glioma cilia. We designed CRISPR/Cas9 gRNAs against two key ciliogenesis genes, *KIF3A* and *ARL13B*. KIF3A is an anterograde motor subunit, without which glioma cilia fail to normally assemble [25,34]. We also targeted ARL13B since the 738 treatment increased the frequency of ARL13B+ cilia (Figure 3B–F). ARL13B also has known roles in cilia growth and stability and in mediating ciliary signaling [28,29]. We generated two ARL13B-depleted clones from patient-derived S7 cells, from which we expanded cell lines that were confirmed to lack ARL13B+ cilia by immunostaining (Figure S5A–J) and ARL13B by Western blot (Figure S5L). We found that these ARL13B/cilia-depleted cell lines proliferated at a slower rate than the parental lines (Figure S5K). We then investigated whether 1215 or 738 reduced the proliferation of these cell lines to the same extent as ARL13B+/ciliated parental cell lines. We treated S7 *ARL13B*-knockout (KO) cells with vehicle, low (500 nM), or high (5 µM) concentrations of 1215 or 738, and measured their proliferation by cell counting five days after treatment. We found that 5-µM 1215 or 738 significantly reduced the proliferation of S7 parental and ARL13B-depleted lines (Figure 4A,B). However, although parental cells exhibited significantly lower proliferation after treatment with 500 nM of either drug, the proliferation of *ARL13B*-KO S7 cells was not affected. We observed similar results with L0 *ARL13B*-KO and *KIF3A*-KO cells (Figure S6A–D). The results indicate that 250- and 500-nM 738 significantly reduced the proliferation of L0 parental cells but not *ARL13B*-KO L0 cells. While 500-nM 738 was still toxic to *KIF3A*-KO cells, 250-nM 738 had no effect (Figure 4C), suggesting that KO of *KIF3A* may lead to extraciliary changes that make cells more sensitive to 738. These results suggest that, as concentrations of HDAC6 inhibitors are decreased, their tumor anti-proliferative effects begin to depend on the presence of glioma cilia.

### 2.6. The Differentiation of Glioma Cells Induced by HDAC6 Inhibition Depends on Primary Cilia

**Figure 5:**
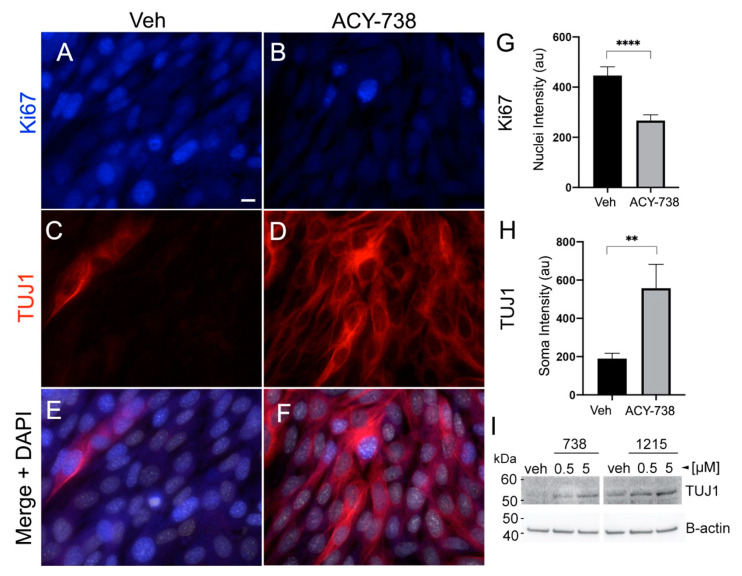
HDAC6 inhibitors promote the differentiation of KR158 cells. KR158 cells were grown to about 70% confluency and treated with vehicle (veh) or 5-µM 738 and fixed 24 h later. (**A**–**F**) Immunostaining for Ki67 (blue) and TUJ1 (red). Bar in A = 10 µm. (**G**) Bar graph is quantification of the background-corrected nuclear intensity of Ki67+ cells. au = arbitrary units. *n* = 60–80 cells/group. (**H**) Bar graph showing quantification of the background-corrected soma intensity of TUJ1. au = arbitrary units. ** *p* < 0.01, **** *p* < 0.001, Student’s *t*-test. (**I**) KR158 cells were grown in six-well plates, treated at about 70% confluency with vehicle (veh), 0.5, or 5-µM 738 or 1215. Cells were harvested after 24 h and 25 µg/lane of protein were analyzed by Western blot with a mouse anti-TUJ1 antibody. ß-actin was used as a loading control.

HDAC6 inhibitors have been reported to reverse the malignant phenotype of glioma cells by forcing their differentiation, as measured by reduction of the proliferative marker Ki67 and enhancement of differentiation markers such as TUJ1 [1,3]. Consistent with these studies, 5-µM 738 reduced the expression of Ki67 (Figure 5A,B,G) and increased the expression of TUJ1 (Figure 5C,D,H) in KR158 cells within 24 h of exposure to 738. Both 1215 and 738 induced a dose-dependent increase in TUJ1 expression (Figure 5I).

**Figure 6:**
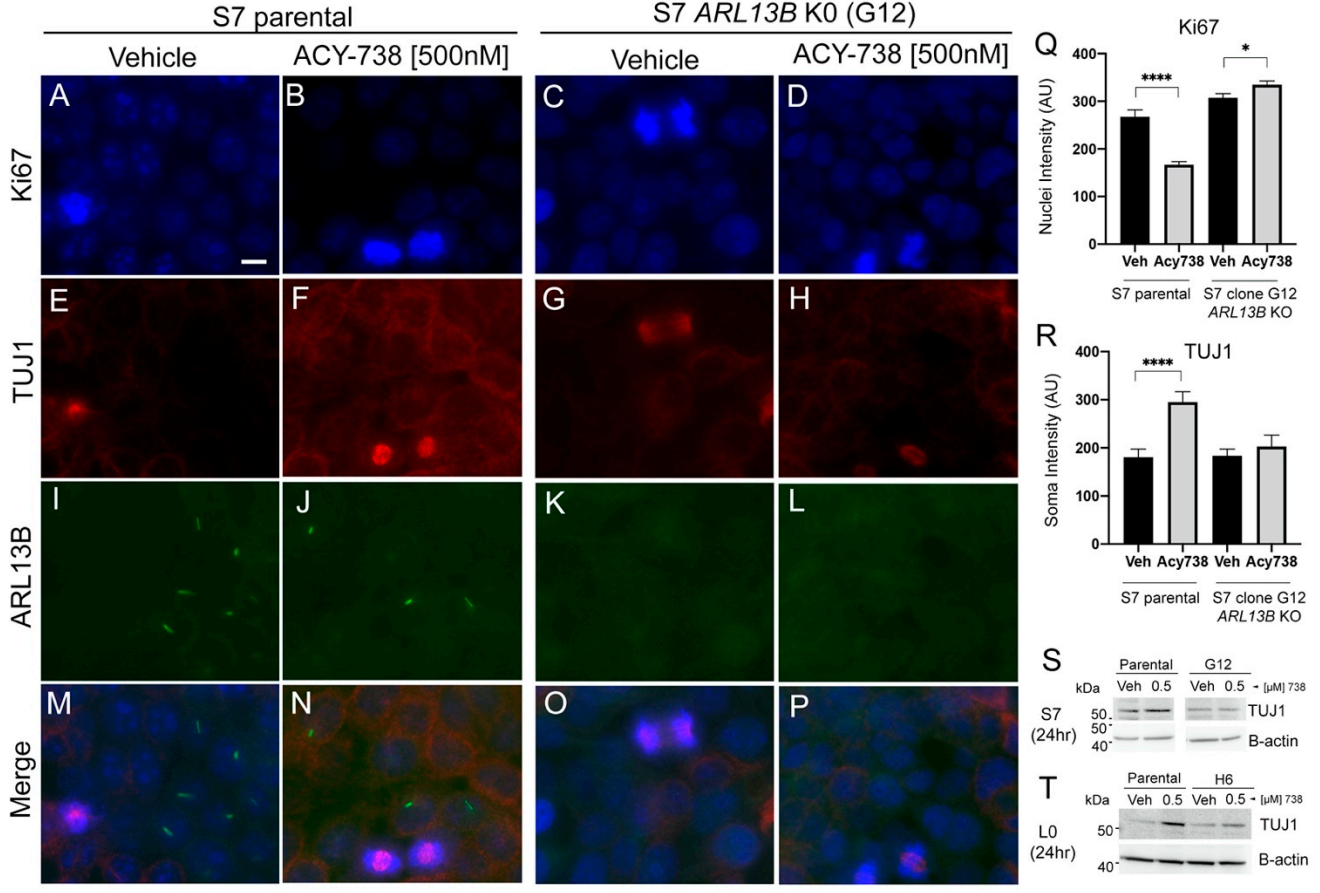
ACY-738 induced changes in Ki67 and TUJ1 are not observed in *ARL13B*-KO, cilia-depleted human glioma cells. (**A**–**P**) S7 parental cells (left two columns) or *ARL13B* KO (clone G12) (right two columns) were grown to ~75% confluency and treated with vehicle or 500-nM 738. Cells were fixed after 24 h and immunostained for Ki67 (**A**–**D**), TUJ1 (**E**–**H**), and ARL13B (**I**–**L**). Merged images are displayed M-P. Scale bar in A = 10 µm. (**Q**) Mean nuclear intensity of Ki67 signal for indicated cell lines and treatments. *n* = 60–80 cells/group. * *p* < 0.05, **** *p* < 0.0001, ANOVA. (**R**) Mean soma intensity of TUJ1 signal for indicated cell lines and treatments. (**S**,**T**) Western blot of S7 (**S**) and L0 (**T**) cell lysates 24 h after exposure to vehicle or 500-nM 738. Blots were probed for TUJ1 and ß-actin as loading controls.

Since 500-nM 738 reduced the proliferation of S7 parental glioma cells but not *ARL13B*-KO/cilia-depleted S7 cells (Figure 5B), we examined whether this decreased proliferation was due to enhanced cell differentiation and whether this effect depended on cilia. We exposed S7 parental or *ARL13B*-KO (clone G12) cells to 500-nM 738 and examined the expression of Ki67 and TUJ1 after 24 h. The 738-treated S7 parental cells displayed reduced Ki67 (Figure 6A,B,Q) and increased TUJ1 (Figure 6E,F,R) immunoreactivity when compared with the vehicle groups. In contrast, the expression levels of Ki67 and TUJ1 appeared similar between vehicle and 738-treated *ARL13B*-KO S7 cells (Figure 6C,D,G,H,Q,R). The absence of a 738-induced increase in TUJ1 in *ARL13B*-KO S7 cells, when compared with parental cells, was also supported by Western blot analyses after exposure to different concentrations of 738 (Figure 6S, Figure S7A). Similar to S7 *ARL13B*-KO cells, L0 *ARL13B*-KO (clone H6) did not show increased TUJ1 after different concentrations of 738 (Figure 6T, Figure S7B). Together, these data suggest that cilia play a crucial role in HDAC6 functions in glioma cell proliferation and that inhibiting HDAC6′s cilia-mediated function promotes the differentiation of tumor cells.

### 2.7. Overexpressing HDAC6 in Glioma Cells Reduces the Levels of Acetylated Alpha-Tubulin but Not Ciliary Length or Frequency of Ciliated Tumor Cells

**Figure 7:**
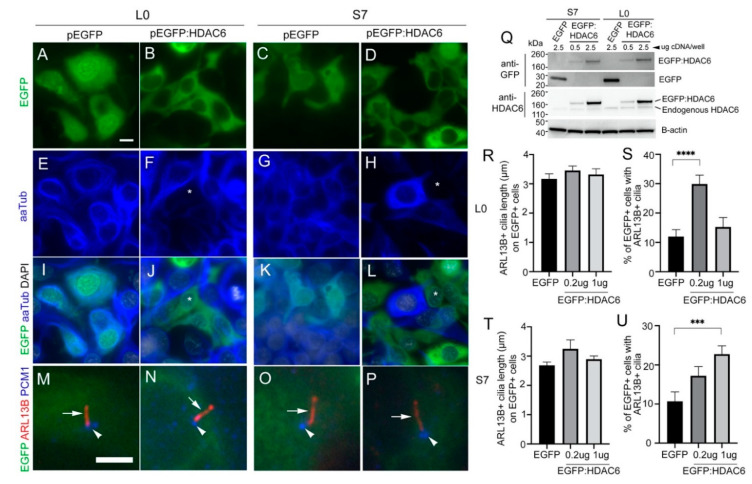
Overexpression of HDAC6 in human glioma cells is not sufficient to reduce cilia length or frequency. L0 and S7 cells were transfected cDNA encoding EGFP or EGFP:HDAC6 and fixed approximately 40 h later. (**A**–**L**) Immunostaining of transfected cells with an antibody for aaTub (blue). AaTub signal was reduced in EGFP:HDAC6+ cells (e.g., asterisks in **F**,**J**,**H**,**L**) compared to neighboring, non-transfected EGFP- cells or EGFP control cells (**I**,**K**). (**M**–**P**) Immunostaining of transfected cells for ARL13B (red, arrow) and PCM1+ (blue, arrowhead). Both L0 and S7 EGFP and EGFP:HDAC6 transfected cells display ARL13B+ cilia. Scale bars (in µm) in A = 10, M = 5. (**Q**) S7 and L0 cells were transfected with the indicated concentration of cDNA encoding EGFP or EGFP:HDAC6, and cell lysates were harvested 40 h later. Western blots were probed for GFP (top two panels), HDAC6, and ß-actin. (**R**–**U**) L0 and S7 cells were transfected with EGFP or two different concentrations of EGFP:HDAC6 cDNA. Bar graphs show cilia length (**R**,**T**) and percentage of the EGFP+ cells with ARL13B+ cilia (**S**,**U**) in the indicated groups. *** *p* < 0.001, **** *p* < 0.0001, ANOVA.

Lastly, we wanted to examine the relationship between cilia frequency in glioma and HDAC6 expression levels. Would increasing HDAC6 levels have the opposite effect of inhibiting its function, through promoting the deacetylation of aaTub and the disassembly of cilia? Following previous studies in which either flag-, HA-, or GFP-tagged HDAC6 was overexpressed in normal human cholangiocytes or RPE1 cells, resulting in reduced cilia length and frequency after 24 h [20,21], we transfected adherent S7 and L0 cells with cDNA expressing EGFP or mycEGFP:HDAC6 construct. After ~40 h, we fixed and immunostained transfected cells for aaTub, ARL13B, and PCM1 (a protein that clusters around ciliary basal bodies and centrioles [34]). First, we found that EGFP:HDAC6 transfected cells displayed weak immunolabeling for aaTub compared to control EGFP-transfected cells or neighboring untransfected cells (Figure 7A–L), a result that validates the expected targeting of aaTub by HDAC6. In control EGFP-transfected cells, cilia were detectable but weakly labeled with aaTub, a possible transfection-related issue that has been shown to affect primary cilia aaTub staining in other studies [35]. Nevertheless, we did not observe cilia loss on these EGFP:HDAC6-expressing cells. Immunostaining for ARL13B and PCM1 showed cilia on both EGFP:HDAC6-transfected L0 or S7 cells (Figure 7M–P). We then tested whether increasing the amount of EGFP:HDAC6 protein by transfecting different concentrations of the cDNA could reduce the length or frequency of these cilia (Figure 7Q). Transfecting fivefold the amount of EGFP:HDAC6 cDNA had no effect on the length of cilia on EGFP:HDAC6+ in L0 (Figure 7R) or S7 (Figure 7T) cells. Further, increasing EGFP:HDAC6 in L0 and S7 cells had either no effect or significantly increased cilia frequency (Figure 7S,U). Thus, our data suggest that overexpression of HDAC6 alone may not be sufficient to promote cilia disassembly on glioma cells and that other factors may be required to drive cilia disassembly.

## 3. Discussion

Previous studies in non-glioma cells have found that the effects of HDAC6 inhibitors on cell proliferation are cilia dependent [22]. More recently, HDAC6 inhibitors have been found to induce the differentiation of glioma stem cells [7,8]. Our study links these two major phenomena in glioma and suggests that the cilium is essential for HDAC6 functions in maintaining the tumor’s proliferative state. We find that blocking HDAC6 function reduces ciliated glioma cell proliferation and promotes their differentiation. We also reveal subcellular differences between the cytosol and cilia in the regulation of alpha-tubulin acetylation shortly after HDAC6 inhibition. Our findings suggest that glioma growth may be slowed dramatically through treatment with HDAC6 inhibitors that hinder the proliferation and promote the differentiation of tumor cells, and that these effects may be highly dependent on HDAC6 expression and cilia frequency in individual tumors.

Generally, GBMs have been characterized as both highly proliferative and containing relatively low frequencies of cilia (~<1 to ~30% of cells) [24,26,36]. The overexpression of HDAC6 reported in GBMs could partially explain these tumor characteristics [1,3], as higher levels of HDAC6 could promote the disassembly of cilia, hence keeping the frequency of ciliated cells low. We were surprised to observe that the induced overexpression of HDAC6 did not have this effect. It is possible that the EGFP-tag on HDAC6 impacted HDAC6′s ability to localize to cilia since we did not observe the same type of clustering at the ciliary base as after endogenous HDAC6 immunostaining. The relationship between HDAC6 and cilia formation/disassembly in tumor cells could also be more complex and depend on other factors that localize or activate HDAC6 at the ciliary base. For example, in hTERT-RPE1 cells, HDAC6 tubulin deacetylase activity is dependent on HDAC6 phosphorylation by Aurora A kinase and HEF1 and their localization to the cilia basal body [20,21]. Thus, HDAC6 promotion of cilia disassembly in glioma cells may require HDAC6 co-activation by other factors.

HDAC6 may nonetheless be functioning through primary cilia to maintain the tumor proliferative state. Since breaking down cilia is required for cell cycle progression [19], and we did not observe cilia on glioma cells in the M phase of the cell cycle (data not shown), HDAC6 inhibitors may arrest glioma cells in a non-dividing, altered ciliated state characterized by a loss or reduction in ciliary aaTub. We found that inhibiting HDAC6 induces a rapid decrease of aaTub in the glioma cell cilia and an increase in the somas, which is a surprising observation since other groups have shown that HDAC6 localizes around the ciliary base and within the cilia [20,21], suggesting that inhibiting its deacetylase function would increase aaTub in the cilia. However, in our experiments, cilia were not readily immunolabeled for HDAC6. It is possible the HDAC6 inhibitors induce significant recruitment of acetyl groups to the alpha-tubulin in the cell bodies, thus depleting them from the ciliary alpha-tubulin. Alternatively, the decrease in ciliary alpha-tubulin acetylation may be associated with glioma cell differentiation. Indeed, differentiated neurons in the brain typically lack aaTub in their cilia (data not shown), unlike their radial glial precursors [37], through mechanisms that are still unclear. Moreover, HDAC6 inhibitors have been recently reported to drive stem-like cells into senescence [7,8] or a differentiated neuronal state [38], which is consistent with the 738-induced increased expression of TUJ1, a marker of neuronal differentiation, that we observed in human and murine glioma cells. While the mechanisms still need to be investigated, our observations show that inhibiting HDAC6 creates a unique and rapid tubulin acetylation change in the cilia that is opposite to the change observed in the cell body, suggesting that HDAC6 may have cilia-specific functions. The significance of this change and how it might affect ciliary signaling require further study.

We found that the proliferation of human glioma lines was sensitive to a wide range of 1215 and 738 concentrations. In contrast, higher concentrations of either drug were required for inhibiting murine KR158 glioma cell growth. Unlike patient-derived glioma lines, KR158 cells are grown adherent in serum-containing media, which may stimulate cell proliferation and suppress the effects of 1215 or 738. After cilia depletion of patient-derived glioma lines, although high concentrations of 1215- or 738-inhibited tumor cell proliferation, this effect was abrogated at low drug concentrations. Blood/CSF concentrations of 1215 and 738 can range from ~70 nM to ~1.9 µM in vivo [13], similar to the low concentrations we used in our in vitro experiments. Thus, tumors characterized by low cilia frequency or structurally aberrant cilia may not be effectively targeted by physiological concentrations or require higher concentrations to be targeted by these HDAC6 inhibitors. In contrast, tumors displaying a high frequency of cilia may be more sensitive to these drugs.

It is unclear whether the HDAC6-inhibitor mediated changes in acetylation of normal astrocyte cilia represent a potential limitation of the use of HDAC6 inhibition for glioma. Most astrocytes are quiescent in the normal brain, but it is unknown whether HDAC6 inhibitors would alter their proliferation in the glioma environment and whether this would impact tumor growth. It is also not clear whether HDAC6 inhibitors disrupt cilia disassembly and normal neural proliferation in the postnatal brain [39,40]. Since HDAC6 inhibitors such as 738 are being explored for their anti-depressive effects in the adult brain [13], there could be a dual advantage in using HDAC6 inhibitors to target rapidly dividing tumor cells in glioma patients battling depression, which often accompanies this cancer [41].

Importantly, our findings on the dependence of HDAC6 signaling on cilia may be relevant to the resistance of glioma to standard-of-care treatments. Indeed, HDAC6 inhibitors have been shown to enhance glioma cell sensitivity to TMZ and radiotherapy [2,5,7,8]. HDAC6 plays a role in promoting autophagy in glioma cells as a mechanism to promote resistance to TMZ [9] and antitumor immunotherapy [4], and HDAC6 inhibitors were recently reported to disrupt the autophagic process at the primary cilia in other cancer types [42]. Furthermore, primary cilia signaling has been reported to play a role in tumor multidrug resistance [43]. Thus, a better understanding of the relationship between HDAC6 and ciliary signaling may lead to strategies that sensitize glioma cells to standard-of-care or novel therapies.

## 4. Materials and Methods

### 4.1. Cell Culture

L0 (high grade), S3 (high grade), and S7(low grade) cell lines were isolated from human gliomas as previously described [24,44,45]. Mouse KR158 glioma cells were derived from a murine grade III anaplastic astrocytoma [46]. L0, S3, and S7 cells were grown as floating spheres and maintained in NeuroCult NS-A Proliferation medium and 10% proliferation supplement (STEMCELL Technologies, Vancouver, BC, Canada; Cat #05750 and #05753, respectively), 1% penicillin–streptomycin (Thermofisher, Waltham, MA, USA; Cat# 15140122), 20 ng/mL human epidermal growth factor (hEGF) (STEMCELL Technologies, Vancouver, BC, Canada; Cat #78006), and 10 ng/mL basic fibroblast growth factor (bFGF) (STEMCELL Technologies, Vancouver, BC, Canada; Cat #78003). For S7 cells, the media was supplemented with 2 μg/mL heparin (STEMCELL Technologies, Vancouver, BC, Canada; Cat #07980). KR158 cells were grown in DMEM ([-] sodium pyruvate; Corning, Corning, NY, USA; Cat #10017CV), 10% heat-inactivated fetal bovine serum (FBS) (Atlanta Biologicals, Flowery Branch, GA, USA; Cat #SH30070.03) and 1% penicillin–streptomycin. All cells were grown in a humidified incubator at 37 °C with 5% CO_2_. When cells reached confluency, or spheres reached approximately 150 μm in diameter, they were enzymatically dissociated by digestion with Accumax (Innovative Cell Technologies, San Diego, CA, USA; Cat #AM-105) for 10 min at 37 °C or TrypLE Express Enzyme (Gibco, Gaithersburg, MD, USA; Cat #12604013) for 5 min at 37 °C. For human cells grown on glass coverslips, the NeuroCult NS-A Proliferation medium was supplemented with 10% FBS.

To generate *ARL13B*-deficient S7 or L0 cells, we transfected floating S7 cells in six-well plates with 5 μg/well of pSpCas9 BB-2A-GFP(PX458) vector containing gRNA targeting human ARL13B (Genscript, Piscataway, NJ, USA; Cat #U994KEE060-2/Q385940) using Lipofectamine 3000. To generate *KIF3A*-deficient L0 cells we followed our previous approach [34], in which we used a CRISPR/Cas9-encoding plasmid containing a GFP reporter gene and gRNA targeting human *KIF3A* [Sigma-Aldrich, St. Louis, MO, USA; CRISPR/Cas-GFP vector (pU6-gRNA-CMV-Cas9:2a:GFP); primer pair ID: HS0000342157; KIF3A gRNA target sequence: GGTCATATTGCAAAAGCGGAGG]. After approximately 3–5 days, we used flow cytometry to sort GFP+ clones in 96-well plates. Viable clones were expanded and screened for ARL13B or KIF3A expression by Western blot and immunostaining, as described below.

Primary neuronal cultures were prepared as previously described [47,48,49]. Briefly, acutely dissociated mouse midbrains from 0–2-day-old C57BL6 male and female pups were isolated and incubated in dissociation medium at 35–37 °C under continuous oxygenation for 60–90 min. Dissociated cells were triturated with pipettes of decreasing bore size (including a punctured fire-polished pipette), then pelleted by centrifugation at 1500 rpm for 3–5 min, and resuspended and plated in the glial medium [47,48,49]. Cells were plated at a density of 100,000 cells/coverslip on 12-mm coverslips coated with 0.1 mg/mL poly-D-lysine and 5 μg/mL laminin and maintained in neuronal media. After 2 h, cells were supplemented with neuronal media (DIV0 composition). Every four days, half of the media was replaced with fresh media. On DIV12, we added vehicle (= volume of DMSO) or 1-µM 1215, then cells were fixed 24 h later in 4% PFA or harvested to generate protein lysates for WB analysis, as described below.

### 4.2. Cell Proliferation Assessment

For cell proliferation assay, cells (5 × 10^4^) were seeded in 500 μL of growth media per well in 24-well plates for each experimental group. After five days, cells were enzymatically dissociated and replaced in 1× phosphate-buffered saline (PBS). Total cell counts were collected using a Bio-Rad TC20 (Bio-Rad Laboratories, Hercules, CA, USA) automated cell counter. Mean cell counts were normalized to vehicle-treated controls and compared statistically using ANOVA.

### 4.3. Immunostaining

For immunocytochemical analyses, cells were enzymatically dissociated and replated in 24-well dishes on uncoated, glass coverslips. Cells were fixed at indicated time points with 4% paraformaldehyde in 0.1 M phosphate buffer (4% PFA). Cells were incubated in blocking solution containing 5% normal donkey serum (NDS) (Jackson Immunoresearch, West Grove, PA, USA; Cat#NC9624464) and 0.2% Triton-X 100 in 1× PBS for 1 h and then incubated in primary antibodies with 2.5% NDS and 0.1% Triton-X 100 in 1× PBS either for 2 h at room temperature (RT) or overnight at 4 °C. Appropriate secondary antibodies (Jackson ImmunoResearch, West Grove, PA, USA) in 2.5% NDS with 1× PBS were applied for 1 h at RT, and coverslips were mounted onto glass slides in Prolong Gold antifade media containing DAPI (Thermofisher, Waltham, MA, USA; Cat# P36935). Stained coverslips were examined under epifluorescence using an inverted Zeiss AxioObserver D1 microscope using a Zeiss 40×/0.95 plan Apochromat air objective. Images were captured and analyzed using Zeiss ZEN software (Carl Zeiss Inc., Thornwood, NY, USA).

For analyses of immunofluorescence staining intensity, areas were traced around the glioma cell cilium, nucleus, or soma, and the mean fluorescence intensity (MFI) was background corrected. The background MFI, measured from an area between the cells, was subtracted from the cilia/nuclei/somas MFI. For soma MFI measurements, a small circle of defined size was placed between the nucleus and outer cell membrane, or within 1–2 µm of the cilium of ciliated cells. For nuclei MFI measurements, a small circle of defined size was placed over DAPI-labeled nuclei. We analyzed cilia, nuclei, and somas from at least three coverslips per group.

### 4.4. Western Blot (WB)

Cells were harvested at indicated time points and lysed in 1× radioimmunoprecipitation assay (RIPA) buffer (Cell Signaling, Danvers, MA, USA; Cat# 501015489) containing 1× protease inhibitor cocktail (Sigma, St. Louis, MO, USA; Cat# P2850), phosphatase inhibitor cocktails 1 (Sigma, St. Louis, MO, USA; Cat# P5726), and 2 (Sigma; Cat# P0044), and 1× phenylmethanesulfonyl fluoride (Sigma, St. Louis, MO, USA; Cat# 93482). A total of 25 to 30 μg of total protein lysate per lane were separated on a 4–12% Bis-Tris gel (Thermofisher; Cat# NP0050). Proteins were blotted onto PVDF membranes using iBlot (program 3 for 8 min; Invitrogen, Carlsbad, CA, USA). Blots were blocked in 5% nonfat dry milk (NFDM) or bovine serum albumin (BSA, Jackson Immuno Research, West Grove, PA, USA; Cat# NC9871802) in 1× tris-buffered saline (TBS) with 0.1% Tween (TBST) for 20 min and then incubated in primary antibodies in 2.5% NFDM or BSA in 1× TBST for 24 h at 4 °C. Blots were then rinsed and probed in the appropriate horseradish peroxidase (HRP)-conjugated secondary antibody (1:10,000; BioRad, Hercules, CA, USA) for 30 min at RT in 2.5% NFDM or BSA in 1× TBST. Finally, blots were rinsed in 1× TBS and developed using an Amersham ECL chemiluminescence kit (Global Life Sciences Solutions USA, Marlborough, MA, USA), and images were captured using an AlphaInnotech Fluorchem Q Imaging System (Protein Simple, San Jose, CA, USA). Selected areas surrounding the predicted molecular weight of the protein of interest were extracted from whole blot images (Figure S8).

### 4.5. Materials

Primary antibodies used for immunocytochemistry (ICC) and WB included mouse anti-aaTub (1:10,000; Sigma, St. Louis, MO, USA; Cat #T6793), rabbit anti-ARL13B antibody (1:5000; Proteintech, Rosemont, IL, USA; Cat #17711-1-AP), mouse anti-TUJ1 (1:2000; R&D Systems, Minneapolis, MN, USA; Cat #NL1195V), chicken anti-Ki-67 (1:5000; Encor, Gainesville, FL, USA; Cat# CPCA-Ki67), chicken anti-GFAP (1:1000; Encor, Gainesville, FL, USA; Cat# CPCA-GFAP), mouse anti-GAPDH (1:10,000; Encor, Gainesville, FL, USA; Cat# MCA-1D4), chicken anti-GFP (1:5000; Abcam, Cambridge, UK; #ab13970), rabbit anti-HDAC6 (1:1000; Sigma, St. Louis, MO, USA; Cat #H2287), rabbit anti-HDAC6 (1:1000; Cell Signaling, Danvers, MA, USA; Cat #7558S), mouse anti-KIF3A (BD Biosciences, San Jose, CA, USA; Cat #611508), and rabbit anti-PCM1 (1:1000; Bethyl Laboratories, Montgomery, TX, USA; Cat# A301-150A). ACY-1215 (APEXbio, Houston, TX, USA; Cat #A4083) and ACY-738 (MedChemExpress, Monmouth Junction, NJ, USA; Cat #1375465-91-0) were dissolved in 100% DMSO (Fisher Scientific, Waltham, MA, USA; Cat # D128-500) to produce a stock concentration of 10 mM. cDNA vectors pCMV-EGFP or pCMV-mycEGFP:HDAC6[#NM_001321225.2] were designed and obtained from Vectorbuilder (Vectorbuilder.com (accessed on 29 March 2021)).

## 5. Conclusions

Overexpressing HDAC6 alone does not appear sufficient to reduce glioma cilia frequency or length. However, glioma cell proliferation is significantly disrupted by HDAC6 inhibition. We show that inhibiting HDAC6 activity increases the number of ciliated glioma cells while reducing the level of acetylated alpha-tubulin in primary cilia. Moreover, low concentrations of HDAC6 inhibitors fail to inhibit the proliferation or induce the differentiation of glioma cells lacking cilia. Our data suggest that HDAC6 interactions with glioma cilia are essential for these tumors to maintain a proliferative state.

## Data Availability

Data supporting the findings within this study are presented within the article and are available from the corresponding author upon reasonable request.

## Supplementary Materials

The following are available online at https://www.mdpi.com/article/10.3390/cancers13071644/s1, Figure S1: ACY1215 and ACY738 treatment induces cell death of patient-derived and murine GBM cells and arrest their growth in the G2/M cell cycle phase. (A–F) Brightfield pictures of patient glioma L0 (A–C) and S7 (D–F) cell lines after five days in culture with vehicle (equal volume of DMSO) or 5-µM 1215 or 738. Bar in C = 200 µm. (G) Sphere diameters of S7 cells after indicated treatments. (H,I) Flow cytometry analysis of L0 cells in G2/M (H) or sub G1 (I) cell cycle phases 24 or 48 h after treatment with vehicle or 1215. (J) Adherent murine KR158 cells treated with vehicle (equal volume of DMSO), 500-nM or 5-µM 1215 or 738. After five days, cells were dissociated and counted. * *p* < 0.05, ** *p* < 0.01, *** *p* < 0.001 (ANOVA). (K) S3 cells treated with DMSO (equal volume) or 5-µM ACY1215. After 5days, spheres were dissociated and cells counted. *** *p* < 0.001, Student’s *t*-test. Figure S2: Expression of HDAC6 in glioma cells. (A–C) Immunostaining of S3 cells for HDAC6 (green, arrowheads) and gTub (red, arrows). Note clusters HDAC6 puncta (arrowheads) around gTub centrioles and ciliary basal bodies (arrows). D–I) Immunostaining of HDAC6 (green) and aaTub (red). Upper (D–F) and lower (G–I) panels show HDAC6 puncta (arrowheads) associated with the aaTub+ axonemes (arrows). (J–R) Three examples of S7 cells immunostained for ARL13B (green) and HDAC6 (red). Nuclei are labeled with DAPI (blue). Arrowheads in each example show clustering of HDAC6 around the base of ARL13B+ cilia. Note that the rabbit anti-HDAC6 antibody used to stain S3 cells was from Sigma, while the rabbit antibody used to stain S7 cells was from Cell Signaling. Scale bars in F and R = 5 µm. Figure S3: Detectable axonemal microtubules in the primary cilium of L0 cell treated with ACY738. (A,B) Adherent L0 cells were treated with 5-µM 738 and fixed 24 h later for TEM analysis. Example of a cilium in A (arrow), magnified in B, showing the longitudinally aligned microtubule arrays inside the axoneme. Figure S4: ACY1215-induced loss of ciliary acetylated alpha-tubulin in normal mouse GFAP+ astrocytes. (A–E) Primary cultures from mouse midbrain were grown for 10–12 days in vitro, treated with vehicle (left panels) or 1-µM ACY1215 (right panels), fixed 24 h later, and immunostained for aaTub (A, red), ARL13B (B, green), and glial fibrillary acidic protein (GFAP) (C, blue). Merged images are shown in D. Arrows point to ARL13B+ cilia whose magnifications were enlarged and rotated in E. Note reduction of aaTub intensity in 1215-treated ARL13B+ cilia. (**F**) Quantification of aaTub intensity in ARL13B+ cilia (veh (*n* = 36 cilia), 1215 (*n* = 66 cilia)). (**G**) Adherent primary cultures derived from fetal mouse brain were treated with vehicle or indicated concentrations of 1215 or 738. Cells were harvested and lysed after 24 h, and protein lysates were Western blotted for ARL13B and aaTub, Figure S5: CRISPR/Cas9-mediated deletion of *ARL13B* in S7 glioma cells leads to loss of cilia and reduced proliferation. (A–J) S7 cells were transfected with CRISPR/Cas9 plasmids targeting human *ARL13B*. After several weeks, clones were isolated, expanded, and screened for ARL13B by immunostaining and Western blot. Examples of clone G12 (B,E,H) and H12 (C,F,I) in which ARL13B+ (or aaTub+) cilia were not observed by immunostaining. Bar in C = 10 µm. (J) Quantification of the percentage of ARL13B+ cilia. (K) Loss of ARL13B also slowed the expansion of the tumor cells in culture. (L) Loss of ARL13B at the predicted MW of ~55kDa in cell lines derived from clones G12 and H12. GAPDH was used as a loading control. *** *p* < 0.001 (ANOVA). Figure S6: CRISPR/Cas9-mediated deletion of *ARL13B* and *KIF3A* on L0 glioma cells. L0 cells were transfected with CRISPR/Cas9 gRNA targeting human *ARL13B* or *KIF3A*. Several weeks after transfection, clones were isolated, expanded, and screened for ARL13B of KIF3A expression by Western blot and immunostaining. (A) Western blot of cell lysates collected from parental, clone D10 (*KIF3A* KO), and clone H6 (*ARL13B* KO). (B–D) Immunostaining for ARL13B and PCM1 in parental, clone D10, or clone H6 cells. Parental cells display ARL13B+ cilia (B, arrow) with PCM1 clustered at the ciliary base. However, in *KIF3A*-KO cells, ARL13B clusters at or around PCM1 puncta, and positive cilia were not observed (C). *ARL13B*-KO cells displayed PCM1 clusters but lacked ARL13B staining (D). Bar in B = 5 µm, Figure S7: ACY738-induced increase in TUJ1 does not occur in *ARL13B*/cilia-depleted cells. (A) Western blot analyses of TUJ1 after 24 h of 250-nM 738 treatment of S7 parental or *ARL13B*-deficient (clone G12) cells. (B) Western blot analyses of TUJ1 after 24 h of 250-nM 738 treatment of L0 parental or *ARL13B*-deficient (clone H6) cells. β-actin was used as a loading control. Figure S8: Original western blot figures.
