# Supplementary material for: HDAC6 Signaling at Primary Cilia Promotes Proliferation and Restricts Differentiation of Glioma Cells
Source: Cancers (Basel). 2021 Apr 1;13(7):1644. doi: 10.3390/cancers13071644 (PMC8036575; doi:10.3390/cancers13071644)
Supplement: Supplementary file 1 [file cancers-13-01644-s001.pdf]

Supplementary Materials: HDAC6 Signaling at Primary Cilia Promotes Proliferation and Restricts Differentiation of Glioma Cells

Ping Shi, Lan B. Hoang-Minh, Jia Tian, Alice Cheng, Reemsha Basrai, Neil Kalaria, Joseph J. Lebowitz, Habibeh Khoshbouei, Loic P. Deleyrolle and Matthew R. Sarkisian

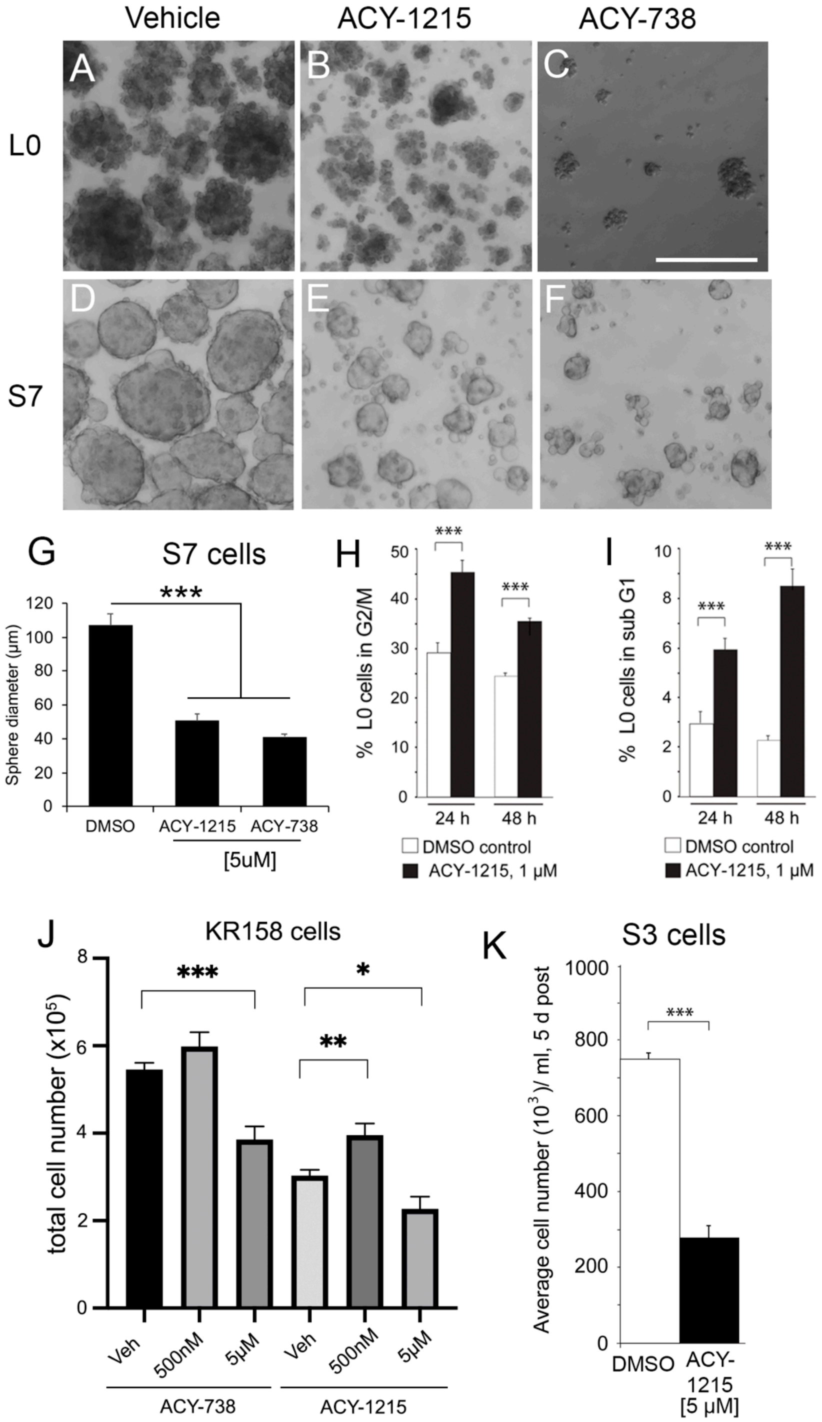

Figure S1. ACY1215 and ACY738 treatment induces cell death of patient-derived and murine GBM cells and arrest their growth in the G2/M cell cycle phase. (**A–F**) Brightfield pictures of patient glioma L0 (**A–C**) and S7 (**D–F**) cell lines after 5

days in culture with vehicle (equal volume of DMSO) or 5-µM 1215 or 738. Bar in C = 200 µm. (**G**) Sphere diameters of S7 cells after indicated treatments. (**H**,**I**) Flow cytometry analysis of L0 cells in G2/M (**H**) or sub G1 (**I**) cell cycle phases 24 or 48 h after treatment with vehicle or 1215. (**J**) Adherent murine KR158 cells treated with vehicle (equal volume of DMSO), 500-nM or 5-µM 1215 or 738. After 5 days, cells were dissociated and counted. * $p < 0.05$, ** $p < 0.01$, *** $p < 0.001$ (ANOVA). (**K**) S3 cells treated with DMSO (equal volume) or 5-µM ACY1215. After 5days, spheres were dissociated and cells counted. *** $p < 0.001$, Student's t-test.

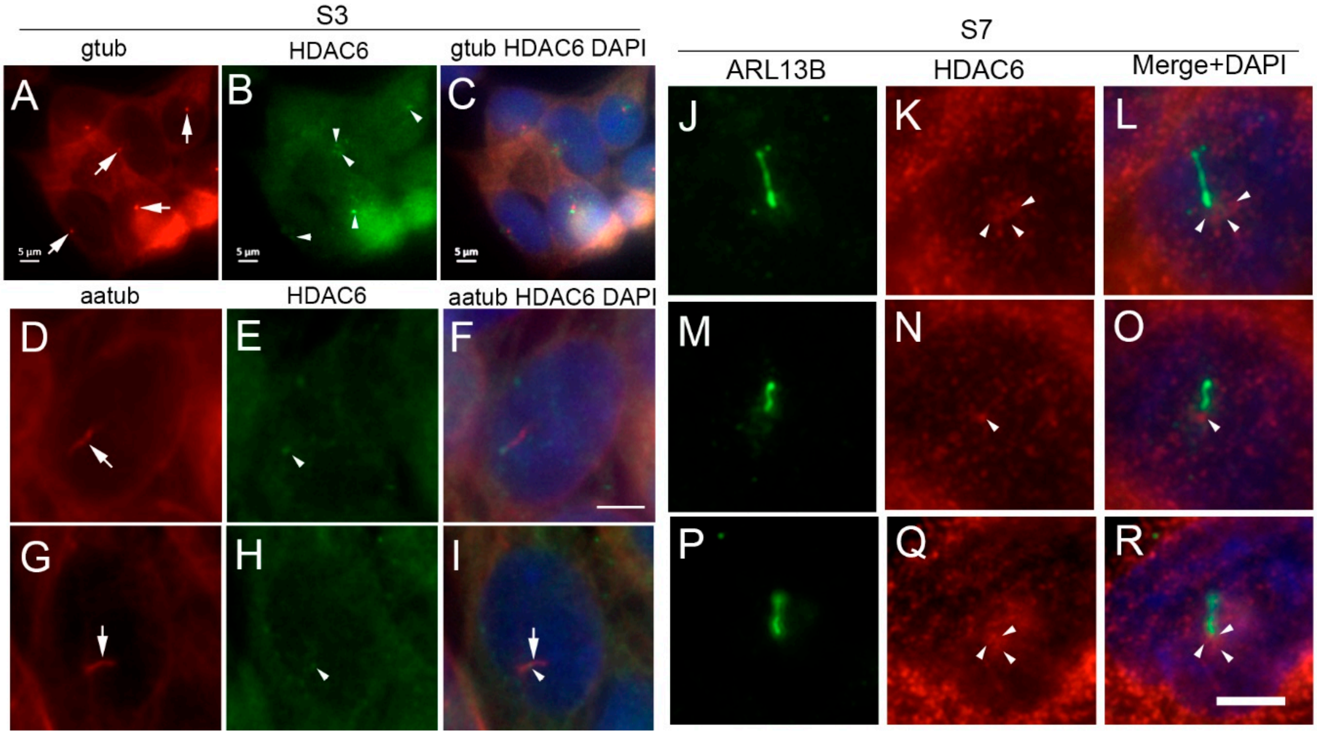

Figure S2. Expression of HDAC6 in glioma cells. (**A–C**) Immunostaining of S3 cells for HDAC6 (green, arrowheads) and gTub (red, arrows). Note clusters HDAC6 puncta (arrowheads) around gTub centrioles and ciliary basal bodies (arrows). (**D–I**) Immunostaining of HDAC6 (green) and aaTub (red). Upper (**D–F**) and lower (**G–I**) panels show HDAC6 puncta (arrowheads) associated with the aaTub+ axonemes (arrows). (**J–R**) Three examples of S7 cells immunostained for ARL13B (green) and HDAC6 (red). Nuclei are labeled with DAPI (blue). Arrowheads in each example show clustering of HDAC6 around the base of ARL13B+ cilia. Note that the rabbit anti-HDAC6 antibody used to stain S3 cells was from Sigma, while the rabbit antibody used to stain S7 cells was from Cell Signaling. Scale bars in F and R = 5µm.

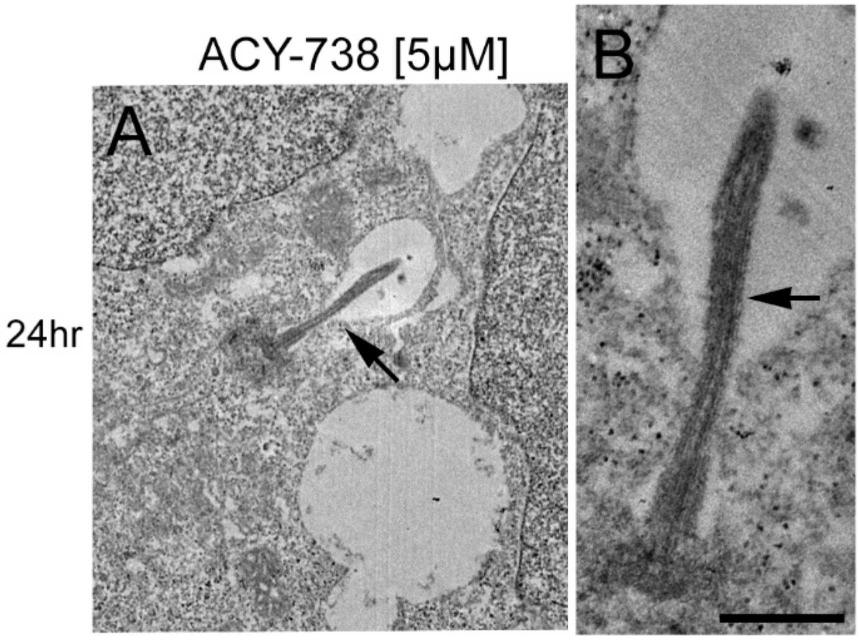

Figure S3. Detectable axonemal microtubules in primary cilium of L0 cell treated with ACY738. (**A,B**) Adherent L0 cells were treated with 5-µM 738 and fixed 24 h later for TEM analysis. Example of a cilium in A (arrow), magnified in B, showing the longitudinally aligned microtubule arrays inside the axoneme.

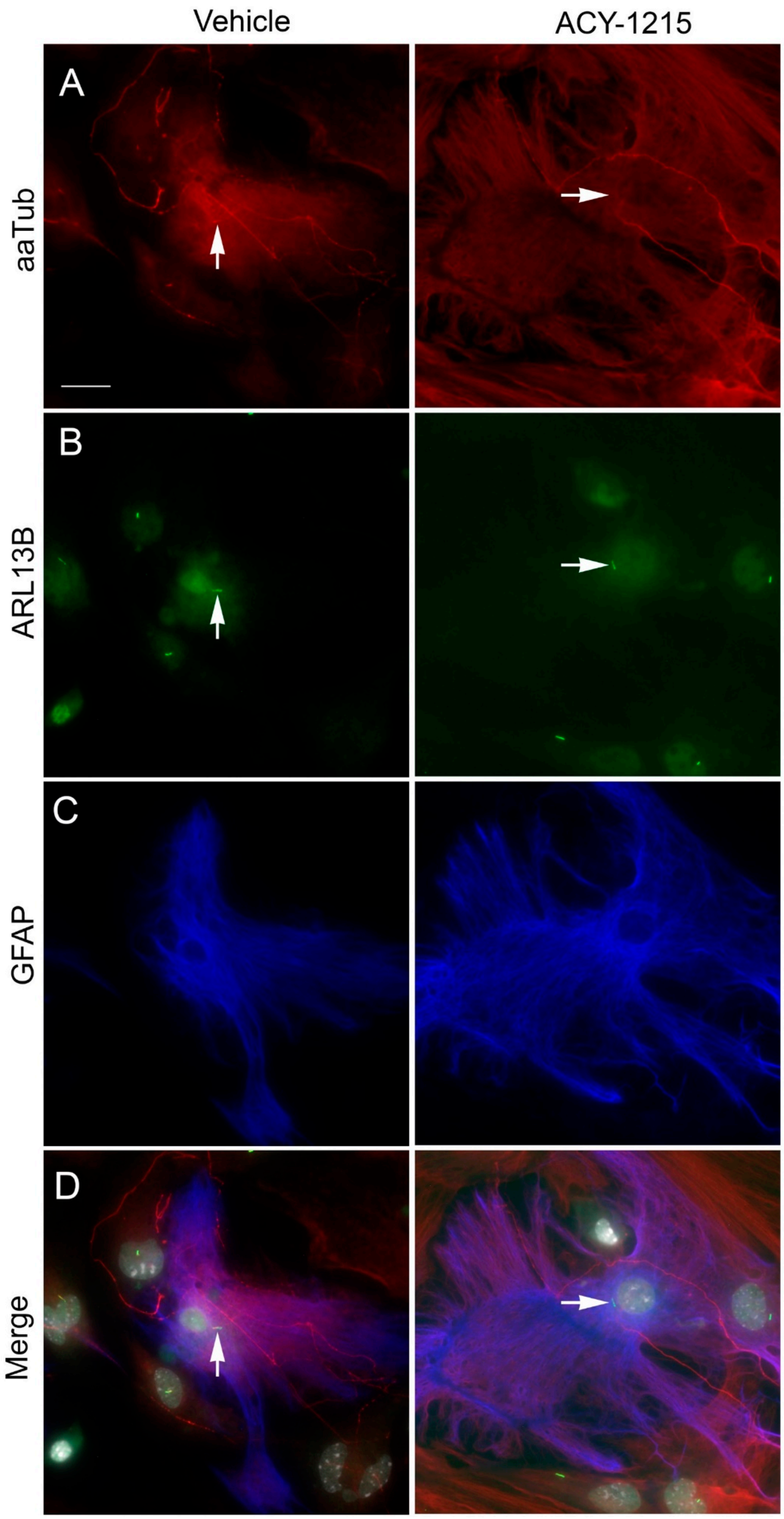
Vehicle
ACY-1215
A
aaTub
B
ARL13B
C
GFAP
D
Merge

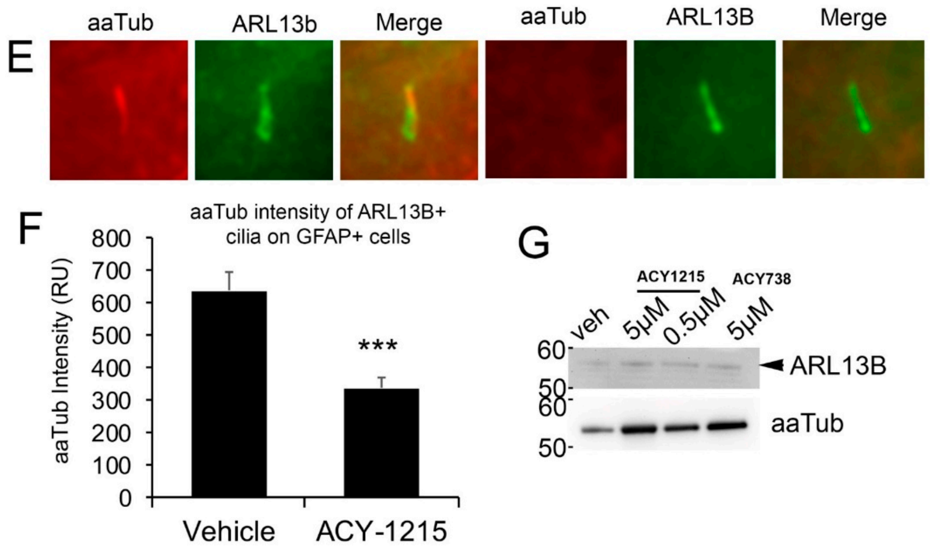

Figure S4. ACY1215-induced loss of ciliary acetylated alpha-tubulin in normal mouse GFAP+ astrocytes. (**A–E**) Primary cultures from mouse midbrain were grown for 10–12 days in vitro, treated with vehicle (left panels) or 1-μM ACY1215 (right panels), fixed 24 h later, and immunostained for aaTub (**A**, red), ARL13B (**B**, green), and glial fibrillary acidic protein (GFAP) (**C**, blue). Merged images are shown in **D**. Arrows point to ARL13B+ cilia whose magnifications were enlarged and rotated in E. Note reduction of aaTub intensity in 1215-treated ARL13B+ cilia. **F**) Quantification of aaTub intensity in ARL13B+ cilia (veh (n = 36 cilia), 1215 (n = 66 cilia)). **G**) Adherent primary cultures derived from fetal mouse brain were treated with vehicle or indicated concentrations of 1215 or 738. Cells were harvested and lysed after 24 h, and protein lysates were western blotted for ARL13B and aaTub.

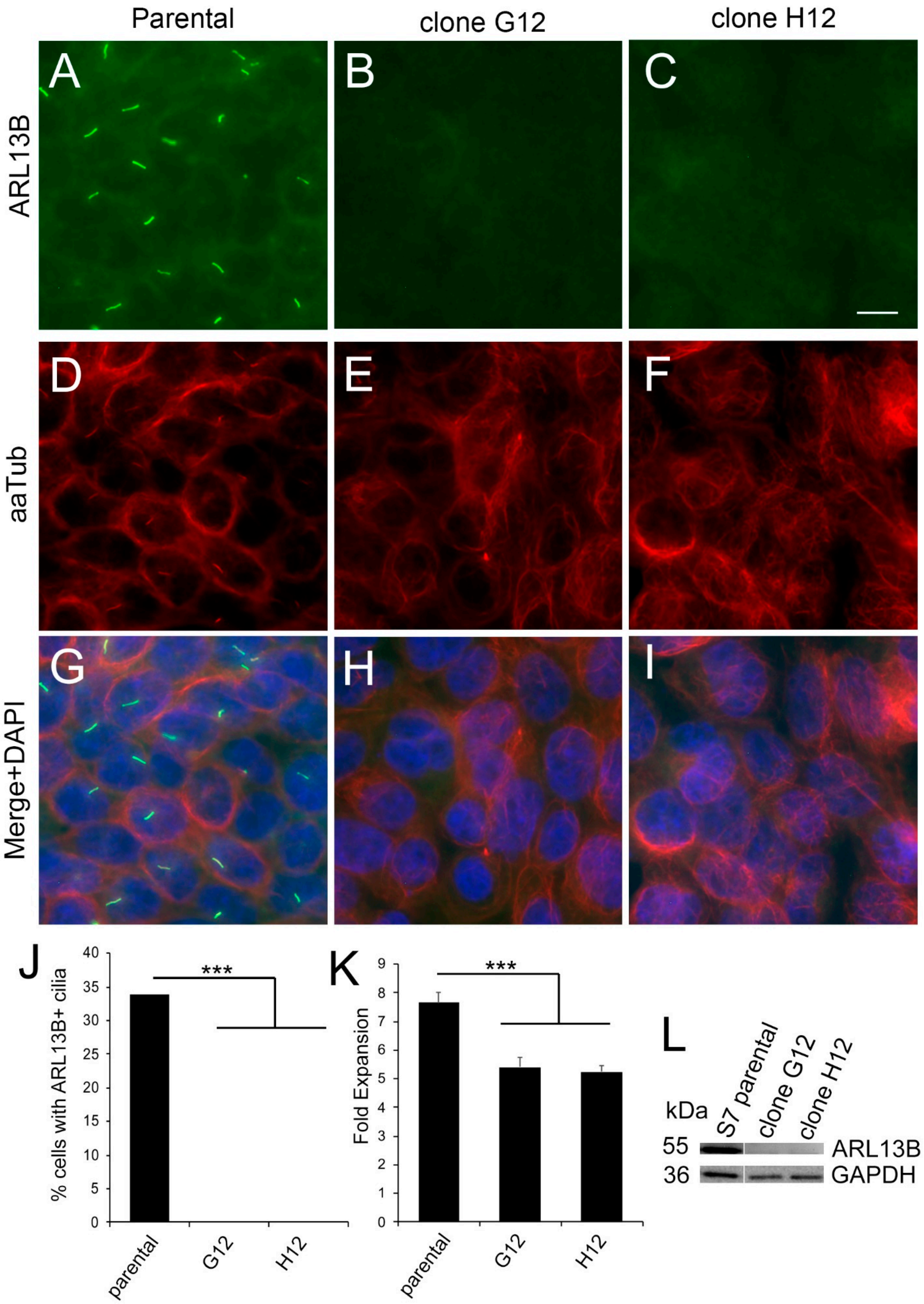

Figure S5. CRISPR/Cas9-mediated deletion of *ARL13B* in S7 glioma cells leads to loss of cilia and reduced proliferation. (**A–J**) S7 cells were transfected with CRISPR/Cas9 plasmids targeting human *ARL13B*. After several weeks, clones were isolated, expanded, and screened for ARL13B by immunostaining and western blot. Examples of clone G12 (**B**, **E** and **H**)

and H12 (**C**, **F** and **I**) in which ARL13B+ (or aaTub+) cilia were not observed by immunostaining. Bar in C = 10 μm. J) Quantification of the percentage of ARL13B+ cilia. K) Loss of ARL13B also slowed the expansion of the tumor cells in culture. L) Loss of ARL13B at the predicted MW of ~55kDa in cell lines derived from clones G12 and H12. GAPDH was used as a loading control. *** $p < 0.001$ (ANOVA).

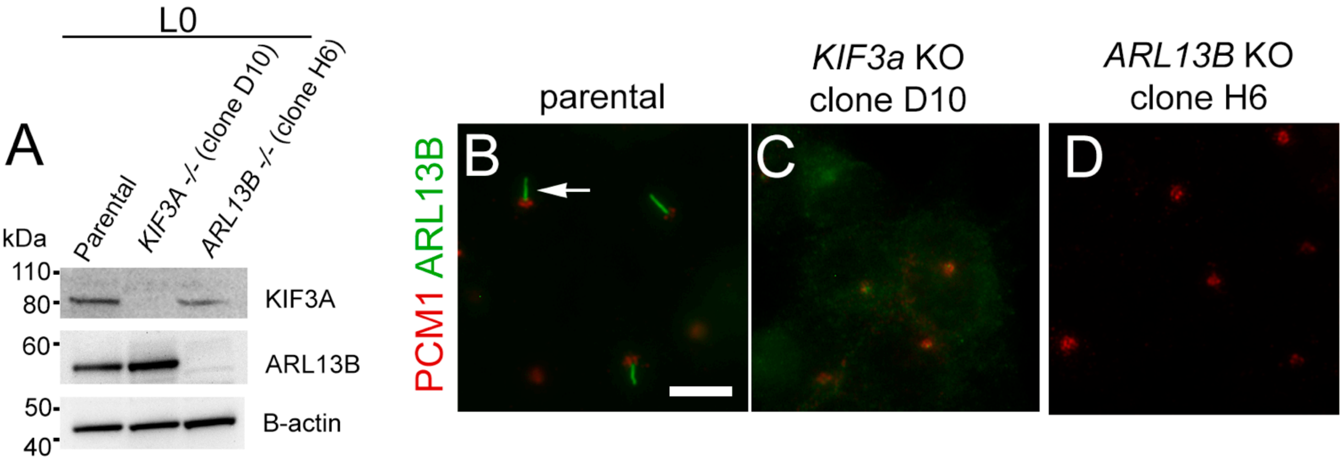

Figure S6. CRISPR/Cas9-mediated deletion of *ARL13B* and *KIF3A* on L0 glioma cells. L0 cells were transfected with CRISPR/Cas9 gRNA targeting human *ARL13B* or *KIF3A*. Several weeks after transfection, clones were isolated, expanded, and screened for ARL13B of KIF3A expression by western blot and immunostaining. **A**) Western blot of cell lysates collected from parental, clone D10 (*KIF3A* KO), and clone H6 (*ARL13B* KO). (**B–D**) Immunostaining for ARL13B and PCM1 in parental, clone D10, or clone H6 cells. Parental cells display ARL13B+ cilia (**B**, arrow) with PCM1 clustered at the ciliary base. However, in *KIF3A*-KO cells, ARL13B clusters at or around PCM1 puncta, and positive cilia were not observed (**C**). *ARL13B*-KO cells displayed PCM1 clusters but lacked ARL13B staining (**D**). Bar in B = 5μm.

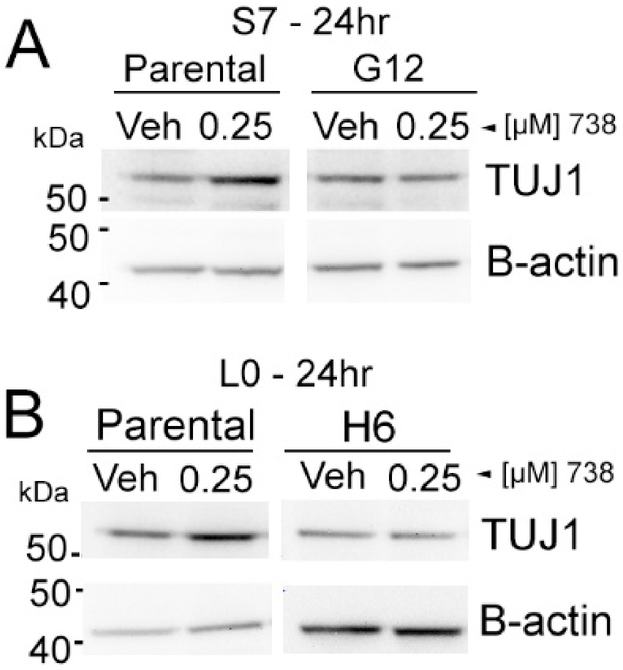

Figure S7. ACY738-induced increase in TUJ1 does not occur in *ARL13B*/cilia-depleted cells. (**A**) Western blot analyses of TUJ1 after 24 h of 250-nM 738 treatment of S7 parental or *ARL13B*-deficient (clone G12) cells. (**B**) Western blot analyses of TUJ1 after 24 h of 250-nM 738 treatment of L0 parental or *ARL13B*-deficient (clone H6) cells. β-actin was used as a loading control.

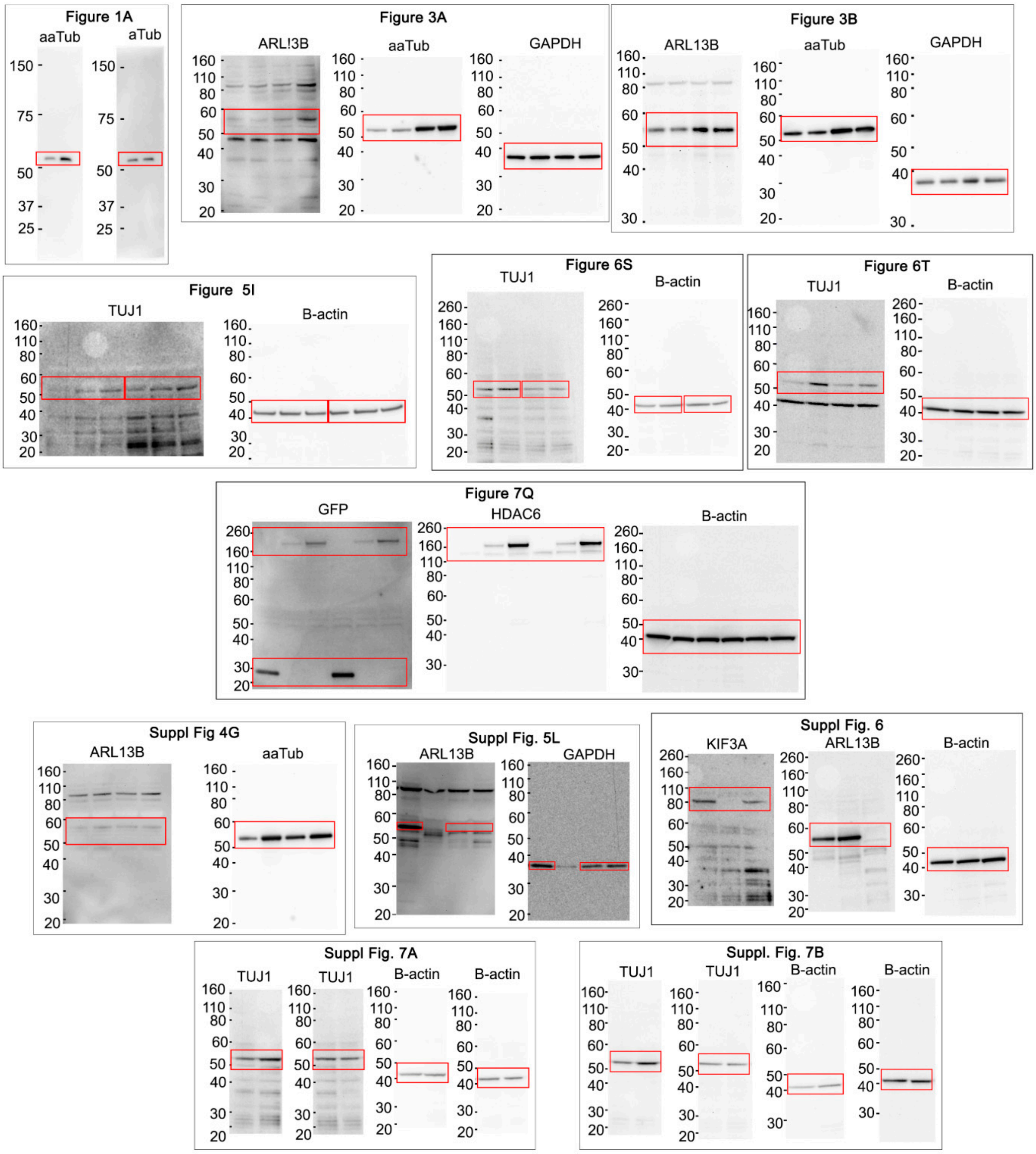

Figure S8. Original western blot figures.
